# Digital Storytelling as a Patient Engagement and Research Approach With First Nations Women: How the Medicine Wheel Guided Our Debwewin* Journey

**DOI:** 10.1177/10497323211027529

**Published:** 2021-07-08

**Authors:** Kendra L. Rieger, Marlyn Bennett, Donna Martin, Thomas F. Hack, Lillian Cook, Bobbie Hornan

**Affiliations:** 1 Trinity Western University, Langley, British Columbia, Canada; 2 University of Manitoba, Winnipeg, Manitoba, Canada; 3 Sagkeeng First Nation, Manitoba, Canada; 4 Pimicikamāk Nīhithawī First Nation, Manitoba, Canada

**Keywords:** aboriginal, arts-based research, Canada, collaborative research, digital storytelling, epistemology, First Nations, Indigenous, patient engagement, qualitative, storytelling

## Abstract

When research is conducted from a Western paradigm alone, the findings and resultant policies often ignore Indigenous peoples’ health practices and fail to align with their health care priorities. There is a need for decolonized approaches within qualitative health research to collaboratively identify intersecting reasons behind troubling health inequities and to integrate Indigenous knowledge into current health care services. We engaged with First Nations women to explore to what extent digital storytelling could be a feasible, acceptable, and meaningful research method to inform culturally safe health care services. This novel approach created a culturally safe and ethical space for authentic patient engagement. Our conversations were profound and provided deep insights into First Nations women’s experiences with breast cancer and guidance for our future qualitative study. We found that the digital storytelling workshop facilitated a *Debwewin* journey, which is an ancient Anishinabe way of knowing that connects one’s heart knowledge and mind knowledge.

There is a long history of using Western approaches in research about Indigenous peoples and of ongoing health care inequities among Indigenous peoples in Canada (Chambers et al., 2018; Hyett et al., 2018; Smith, 2012). Scholars increasingly assert that these two issues are interrelated, and that troubling health care inequities are perpetuated and exacerbated by how new knowledge is developed (Goodkind et al., 2015; Jull et al., 2018; Kendall et al., 2011). Knowledge developed through Western approaches alone can overlook subjugated perspectives and health care practices (Reimer-Kirkham & Anderson, 2002) and fail to align with Indigenous peoples’ health care priorities. For example, in Canada, significant survival disparities exist between First Nations people and settlers for most common cancers, including breast cancer (Horrill et al., 2019; Withrow et al., 2017). Among First Nations women living in Manitoba, breast cancer mortality has increased, whereas it has decreased for all other women (Decker et al., 2016). In addition, First Nations women’s incidence rate of breast cancer is increasing and they are significantly more likely than other Manitoban women to be diagnosed with advanced breast cancer. First Nations women living in Northern communities are more at risk of a later-stage diagnosis than those who live in urban or rural areas (Decker et al., 2016). There is a need to employ decolonizing Indigenous research approaches to identify the intersecting reasons behind these health disparities and to integrate Indigenous knowledge into current health care services (Bennett & Blackstock, 2006; Krusz et al., 2020).

The arts can provide an alternative, multimodal language for expressing life’s experiences (Martin et al., 2018; Rieger et al., 2014, 2018). They provide a container for holistic knowledge as they integrate the emotional, physical, mental, and spiritual aspects of life, which is congruent with Indigenous values. Storytelling has for long been used by Indigenous communities to share knowledge and is increasingly being used as a qualitative research approach by Indigenous peoples and settlers to privilege Indigenous perspectives (Bennett, 2016; Iseke, 2013; Loppie, 2007). Digital storytelling is an expressive arts process that involves participants creating a short video using various multimedia materials (e.g., narrative, photos, video clips, and music) to share a personal story (Bennett, 2016; Lambert, 2013; Rieger et al., 2018).

The purpose of this patient engagement project was to collaborate with First Nations women to explore to what extent digital storytelling could be a feasible, acceptable, and meaningful research method to inform culturally safe health care services. In this article, we articulate how we authentically engaged with First Nations women in a digital storytelling workshop about their breast cancer experiences to explore this method for use in future research projects. We propose that a digital storytelling workshop, guided by the Medicine Wheel (Dapice, 2006; Jeannotte, 2017; LaFever, 2016), can facilitate a *Debwewin* journey, which is a journey of connecting mind and heart knowledge to arrive at one’s personal truth (Gehl, 2012), and create an ethical space for authentic patient engagement and research (Greenwood et al., 2017).

## Background

To address the harms of the past and move forward toward reconciliation, research with Indigenous peoples needs to be respectful and collaborative (Canadian Institutes of Health Research, Natural Sciences and Engineering Research Council, & Social Sciences and Humanities Research Council, 2018). In addition, the Truth and Reconciliation Calls to Action (Truth and Reconciliation Commission of Canada, 2015) and the Missing and Murdered Indigenous Women and Girls Report Calls to Justice (National Inquiry Into Missing and Murdered Indigenous Women and Girls, 2019) recommend the integration of Indigenous knowledges and practices into health care. Furthermore, there have been calls for patient engagement in health care research (Canadian Institutes of Health Research, 2015). Patient engagement occurs when patients meaningfully and actively collaborate in the governance, planning, and conduct of research (Canadian Institutes of Health Research, 2019) and improves the relevance of research findings and knowledge translation initiatives (Bird-Naytowhow et al., 2017; Liebenberg et al., 2017).

Engagement with Indigenous peoples is critical for integrating Indigenous knowledges and practices into health care systems to address health inequities (Jull et al., 2018; Krusz et al., 2020). But authentic patient engagement is challenging, and even more so when there is distrust of Western research institutions and researchers because of a long history of colonization, oppression, criminalization of cultural practices, and structural racism (Bird-Naytowhow et al., 2017; Greenwood et al., 2017). Indigenous patients generally, and First Nations women specifically, have often not been valued as collaborators in research processes (Bird-Naytowhow et al., 2017), resulting in research being done *on* them, rather than *with* them, which continues to reinforce and perpetuate colonization (Bird-Naytowhow et al., 2017; Jull et al., 2018). Thus, finding meaningful and culturally safe ways of engaging *with* First Nations people is crucial to ethical, relevant, and meaningful research.

The principles of cultural safety and humility offer a way forward for researchers. A cultural safety lens acknowledges that the current health care inequities among Indigenous peoples are rooted in a colonial history and shaped by power imbalances (Browne et al., 2009; Horrill et al., 2018). This perspective calls settlers to enact cultural humility to foster transformed environments where people feel safe receiving care and sharing their stories (Browne et al., 2009; First Nations Health Authority, 2020; Horrill et al., 2018). Cultural humility is “a process of self-reflection to understand personal and systemic biases and to develop and maintain respectful processes and relationships based on mutual trust” and “involves humbly acknowledging oneself as a learner when it comes to understanding another’s experience” (First Nations Health Authority, 2020, p. 7). These principles guide us to move away from positioning researchers operating from Western perspectives as experts toward finding new ways of relating that support reciprocity and reconciliation (Bird-Naytowhow et al., 2017; Greenwood et al., 2017).

Collaborative research approaches are needed to move toward respectful relationships, but they must be grounded in Indigenous values and ways of knowing for true transformation in research to occur (Bird-Naytowhow et al., 2017; Greenwood et al., 2017; Kendall et al., 2011; Kwaymullina, 2016). Research and engagement approaches based on Indigenous philosophical assumptions acknowledge and prioritize spirituality and well-being of the whole person, interconnectedness of all the aspects of life and with all living things, relational knowledge development, and relational accountability to communities (Bird-Naytowhow et al., 2017; Goodkind et al., 2015; Kovach, 2010; Loppie, 2007). Bird-Naytowhow and colleagues (2017) argue that “ceremonies of relationship” (p. 1) are needed to meaningfully engage with Indigenous peoples, which they describe as “moments where interactions and exchanges among people for the purposes of knowledge generation (i.e., research) were imbued with ceremonial and spiritual significance” (p. 2) and are focused on relationship building.

We propose a digital storytelling workshop as holding potential for creating decolonized spaces for patient engagement and research (Greenwood et al., 2017). This approach can facilitate researchers’ abilities to process and enact cultural humility, and provide a relational and integrated way of knowing. Digital storytelling helps researchers to learn from patients’ complex experiences and listen deeply, by giving people space to tell their stories and tapping into creative and culturally appropriate ways to illuminate contextual knowledge (Thorne, 2019).

Arts-based, participatory approaches are increasingly being recognized as congruent with Indigenous ways of knowing and valued for elucidating subjugated perspectives and raising consciousness for social change (Drew et al., 2010; Goopy & Kassan, 2019; Hammond et al., 2018; Yi-Frazier, 2015). Goopy and Kassan (2019) used an arts-based engagement ethnography approach in working with a hard-to-reach community and found that it opened new pathways to knowledge development, which shifted the power differential away from the expert researcher, who is usually well-versed in language-centric research approaches, and toward the participant. Scholars describe how storytelling methods can privilege Indigenous patients’ voices, engage participants in culturally relevant and relational ways, incorporate the healing qualities of the arts, and foster an understanding of Indigenous knowledge to guide changes in health care (Archibald et al., 2019; Hammond et al., 2018). Digital storytelling is a participatory arts-based research method (Bennett, 2016; Sitter et al., 2020), which typically involves participants selecting a story that they think is important to share; engaging in group discussions about their stories; writing and recording a narrative; creatively adding multimedia materials to create a compelling, short video; and sharing their video with others (Lambert, 2013; Sitter et al., 2020). In a research study, participants will usually create their digital story in a 2- or 3-day workshop, which includes story circles, and meet with the researcher to talk about the meaning of the story to them (Goodman, 2019; Paterno et al., 2019; Williams et al., 2017). The stories can also be used to share the study findings with patients, health care providers, policy makers, and academics. In previous research, digital storytelling has engaged the community, illuminated issues of importance to participants, meaningfully captured participants’ complex experiences, and provided an engaging mode to share research findings (Bennett, 2016; Fontaine et al., 2019; Goopy and Kassan, 2019; Gubrium et al., 2016; Hammond et al., 2018).

We believe it is important to share experiences with decolonizing methods in patient engagement to inspire dialogue and learn from each other. To that end, in this discussion article, we will share about how we engaged with First Nations women in a digital storytelling workshop, describe how the Medicine Wheel (Dapice, 2006; Jeannotte, 2017; LaFever, 2016) guided our workshop, and discuss how the workshop facilitated a *Debwewin* journey (Gehl, 2012) and why that was important. We collaborated on this article and wrote from diverse perspectives. One of our academics is an Indigenous scholar who specializes in Indigenous knowledges, and three are settlers. All of the academics have advanced graduate degrees. We also collaborated with an Indigenous Elder in the development of the workshop. All of the patient representatives were First Nations women who had experienced breast cancer and some are coauthors on this article. We are aware that First Nations peoples are distinct groups and that the knowledge developed in this study cannot be generalized to all First Nations peoples in Canada. However, we anticipate that this article will add to the conversation of how to meaningfully and respectfully engage with First Nations people about their health and illness experiences, drawing on threads that are common to First Nations peoples, such as the importance of cultural safety, interconnectedness, and relationality, and inspire others to consider storytelling approaches.

## A Digital Storytelling Workshop as a Patient Engagement and Research Approach in Qualitative Research

Our research team was interested in conducting a qualitative research study to explore First Nations women’s experiences of breast cancer with digital storytelling. We viewed our project as occurring in multiple phases. For this first phase, we received a patient engagement grant that allowed us to collaborate with First Nations women to explore to what extent digital storytelling could be a feasible, acceptable, and meaningful research method to inform culturally safe health care services. The objectives of our digital storytelling workshop were to explore Indigenous women’s meaning and conceptualizations of their lived breast cancer experiences to (a) identify factors shaping their health care experiences, (b) identify priority research questions to First Nations women who have experienced breast cancer, (c) assess whether the research design and proposed methods are culturally safe and how we might exercise cultural humility in learning from these women, and (d) explore the feasibility and acceptability of proposed recruitment and study procedures. It was our hope that, through this engagement initiative, we would develop a relational network of Indigenous peoples and settlers for future patient-oriented research.

### Theoretical Perspectives

A Two-eyed Seeing perspective, a framework for research engagement, and the Medicine Wheel guided our work. The Two-eyed Seeing perspective proposes that both Indigenous and Western ways of knowing are important for knowledge development and understanding (Bartlett et al., 2012; Martin, 2012). Thus, each worldview provides important and legitimate understandings, and knowledge is located in a both/and perspective rather than an either/or perspective. As our team consisted of research partnerships between Indigenous people and settlers, a Two-eyed Seeing approach enabled us to bring together an interdisciplinary research team who offered distinct, valuable perspectives to the project.

The Strategy for Patient-Oriented Research (SPOR) patient engagement framework (Canadian Institutes of Health Research, 2015) views patient engagement in research as existing on a spectrum from consultation, to involvement, to collaboration, to directing the research, and we aimed to engage in a collaborative level of engagement with our patient representatives. A collaborative level of engagement involves actively partnering with patient partners in every aspect of the research process. It also includes shared decision-making between researchers and patient partners, and acknowledges and addresses power imbalances. In this first phase of our work, we hoped that our collaborative engagement with patient partners would reveal whether digital storytelling methods are acceptable and culturally appropriate, enable us to cocreate our future qualitative study, and elicit new research ideas based on their needs and priorities.

**Figure 1.**
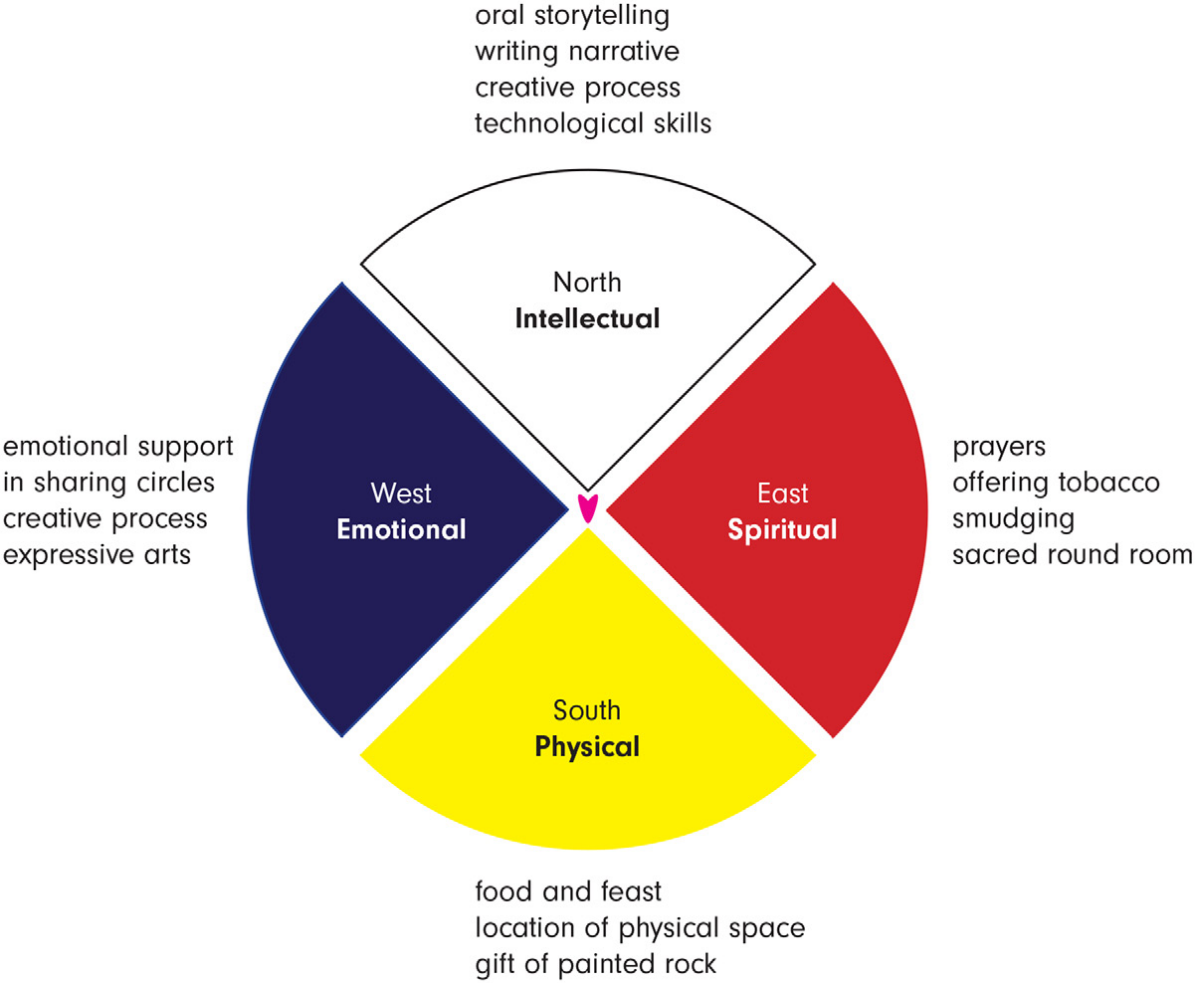
Attending to the four directions in the digital storytelling workshop.

The Medicine Wheel teachings (Dapice, 2006; Jeannotte, 2017; LaFever, 2016) provided a conceptual framework to guide the digital storytelling workshop and to create a culturally relevant and ethical space for engagement (Greenwood et al., 2017). There is some variation in the representation and teachings of the Medicine Wheel, but it is considered a sacred teaching, or set of teachings, which is broadly used across Turtle Island (North America). A common thread between the variations is the ongoing interconnectedness and interrelatedness of all aspects of life, and the need for balance among the different aspects. The medicine of the four directions (east, south, west, and north) are typically represented by four different colors (see Figure 1). These directions can represent many different concepts, including the four aspects of human nature that guided our workshop: mental, emotional, physical, and spiritual. The Medicine Wheel is a symbol of wholeness, which gives equal importance to the four directions and the development of all aspects of human nature to achieve health. It visually depicts the balance of all four elements as being essential to well-being and imbalance as the root of disease (Dapice, 2006; Jeannotte, 2017; LaFever, 2016). Journeying around the Medicine Wheel can facilitate a *Debwewin* journey, which is when one’s head and heart knowledge connect to find one’s truth (Gehl, 2012).

## Method and Data

Our collaborative strategies to engage with our patient partners in the first phase included a 2-day digital storytelling workshop, facilitated by Drs. Bennett and Rieger, and three talking circles throughout the digital storytelling workshop to discuss First Nations women’s experiences of breast cancer, perceptions of what knowledge needs to be developed to improve services, and feedback on the digital storytelling workshop. As this project was a patient engagement initiative, we did not collect, store, or analyze any participant data or need ethical permission to do so. To respect the ethical principles of Indigenous ownership, control, access, and possession (OCAP; First Nations Information Governance Centre, 2019; Phillips-Beck et al., 2019), we employed several strategies. Our shared work was co-led by a settler and an Indigenous scholar. To plan our patient engagement workshop, we offered tobacco and received guidance from a First Nations Elder. We also consulted with leaders in the Indigenous research community who, along with the Elder, reviewed our talking circle questions and provided feedback. This initiative resulted in developing important partnerships with Indigenous patients and community members, which has enabled our ongoing research to be authentically cocreated with a diverse team of Indigenous peoples and settlers (Rieger et al., 2020).

Through connections at a local cancer center and a university, we invited three First Nations women with experiences of breast cancer to participate as patient representatives in our digital storytelling workshop. Inclusion criteria for participation in the workshop were being aged above 18 years, having had a breast cancer diagnosis (primary or recurrent breast cancer), being more than 1-year post major treatment, living in Manitoba, and being willing to participate. We offered honorariums to thank our patient partners for their time.

Our 2-day digital storytelling not only incorporated the conventional format of a digital storytelling workshop, but also included offering tobacco to the participants, opening with a prayer, smudging, three talking circles, and a feast. The workshop included a mix of group sharing and more solitary focused work, as participants chose and worked on a story that they felt was important to share about their breast cancer experiences. See Supplemental File 1 for an outline of the workshop. Drawing on the guidance of StoryCenter (Lambert, 2013), creating the digital stories encompassed an expressive arts creative process that involved (a) choosing the story that they wanted to tell, (b) writing their narrative of approximately 300 to 500 words, (c) recording it digitally, and (d) working on an iPad or laptop computer to create a short video that combined personal photos (~10–15 photographs) and favorite music with their recorded narrative. Participants had much choice in how they told their story. The two workshop facilitators provided technological support, which included teaching participants how to use the iMovie program on an iPad, record one’s narrative, upload and add photos, and edit their videos. One-on-one support was also provided to participants depending on their comfort level with the iMovie program and video editing. The participants’ solitary creative work was interspersed with three talking circles, social times, and meals, and our workshop ended with a viewing of all the videos. See Table 1 for exemplar questions that we asked during the talking circles. A talking circle is an Indigenous tradition in which participants sit in a circle and take the time they need to share, uninterrupted, to ensure that all members are heard (Dylan, 2003). It is slightly different from traditional sharing circles which are viewed as sacred (Tachine et al., 2016), and some believe it is inappropriate to record for research purposes. The talking circles fostered relationships and trust, and provided an opportunity for participants to share their perspectives on the issues emerging from creating and listening to the digital stories and their experiences of the workshop to inform our future research study.

**Table 1.** Talking Circle Exemplar Questions.

First Talking Circle Question Examples
1. What helped you to cope and heal throughout your breast cancer experiences?
2. What was the most challenging for you in dealing with your diagnosis and treatment?
Second Talking Circle Question Examples
1. How do people talk about breast cancer in your community?
2. How do you know when a breast cancer screening program or a breast cancer treatment program is/is not culturally safe?
Third Talking Circle Question Examples
1. What are the most important questions to ask about First Nations women in a future study?
2. Please describe your thoughts on the digital storytelling workshop.
3. How could this workshop be improved?

The traditional digital storytelling workshop was adapted in several ways by an Indigenous scholar and was opened with a prayer and the University of Manitoba (2019) land acknowledgment:

The University of Manitoba campuses are located on original lands of Anishinaabeg, Cree, Oji-Cree, Dakota and Dene peoples, and on the homeland of the Métis Nation. We respect the Treaties that were made on these territories, we acknowledge the harms and mistakes of the past, and we dedicate ourselves to move forward in partnership with Indigenous communities in a spirit of reconciliation and collaboration.

We incorporated Indigenous elements, such as offering tobacco ties, smudging, talking circles, and a feast, to ensure that the workshop was culturally relevant and safe for the women. We offered a tobacco tie at the beginning of the workshop to all participants, as a culturally appropriate and respectful way of asking for the women’s assistance and support in this work. Tobacco is sacred in Indigenous communities and tobacco ties are made by taking a small pinch of ceremonial, organic tobacco and placing it on a small square of cloth, and then forming a small, tied sachet (Wilson & Restoule, 2010). We placed the tobacco tie between us and the patient representative, and specifically asked them to share their story and experiences with us, and all three women picked up the tobacco in agreement (Wilson & Restoule, 2010). We also held a purification (sometimes referred to as smudging) at the start of each day, which is an Indigenous tradition that involves the burning of sacred medicines in a shell or small bowl, such as sweetgrass, sage, and/or cedar, to cleanse a person or place of negative thoughts and create a positive mind-set (University of Manitoba, 2021). The smoke from the burning medicine is viewed as carrying our prayers to the Creator. When participants shared their digital stories with each other, we held a feast with traditional foods and offered a painted rock as a gift to each participant, which Dr. Bennett had gathered from the land and painted with a specific, meaningful symbol for each participant. Rocks hold special meaning for many Indigenous peoples and are viewed as ancient ancestors with spirit. Rocks, according to Ojibwa ontology, are known to be imbued with spirit and provide teachings and instructions on how humans can live well together (Christensen & Poupart, 2012; Hallowell, 2010). The painted rocks were seen as a co-creation of the Creator and a human artist, which echoes processes in our workshop. Our workshop was held in a university facility, located in the center of an urban location highly populated by Indigenous peoples, in which we were able to utilize a sacred round room for smudging. To support businesses owned by Indigenous peoples, our meals were catered by an Indigenous-owned restaurant that serves dishes rooted in First Nations cuisine. To attend to aesthetic elements, Dr. Bennett created a logo (see Supplemental File 1) for our communication documents. We also had culturally relevant psychosocial resources available should a participant become distressed when creating or telling their story.

Although we do not have ethical permission to share the details of what we learned about First Nations women’s experiences of breast cancer in this patient engagement initiative, we can share that our conversations were profound and provided deep insights into First Nations women’s experiences with breast cancer. The women told stories that illuminated the strengths that they drew upon to find healing and also the challenges and racism they experienced within the health care system. In addition, the women provided critical advice for our future qualitative study and how to further adapt digital storytelling for future work together.

From our perspectives, this workshop authentically engaged First Nations women with breast cancer as it was guided by the Medicine Wheel and attended to all aspects of life. The workshop did not just involve intellectual engagement, the traditional Western academic approach, but also engagement with emotional, spiritual, and physical aspects of life (see Figure 1). We attended to intellectual aspects of life through engaging in the intellectual elements of oral storytelling, writing a narrative, and developing technological skills on iPads. To attend to the spiritual aspects of life, we incorporated prayers, offering tobacco, smudging with traditional medicines, and meeting in a sacred round room. To attend to the physical, we provided delicious food and held an Indigenous feast, held the workshop at a meaningful location in the city, and gave each participant a gift of a painted rock that had been created by Dr. Bennett. To attend to the emotional, we offered each other emotional support in the talking circles and used the creative process of expressive arts (digital storytelling) that provided an emotional language to express cancer experiences and perceptions.

## Discussion: Digital Storytelling as a *Debwewin* Journey

By attending to the four aspects of life, we believe that the digital storytelling workshop facilitated a *Debwewin* journey. A *Debwewin* journey is an ancient Anishinabe way of knowing that connects one’s circle of heart knowledge and circle of mind knowledge (Gehl, 2012). It produces one’s *Debwewin*, which is “a personal and holistic truth that is rooted in one’s heart” (Gehl, 2012, p. 53). Gehl (2012) wrote about the *Debwewin* journey and methodology, which she learned from Anishinabe elders and traditional knowledge holders, and we have been guided in its application by Dr. Bennett. Finding one’s *Debwewin* enables one to speak from the heart (Gehl, 2012), which is often lacking in tokenistic patient engagement initiatives.

From this perspective, there are two circles of knowledge: the circle of the heart and the circle of the mind (Gehl, 2012), and knowledge is located in the connecting of the two. Knowledge of the mind or heart alone is viewed as incomplete knowledge (Gehl, 2012) as intelligence is not seen as purely cerebral. The circle of the heart encompasses emotions and spirit (Gehl, 2012) and the circle of the mind encompasses what we would typically think of as intellectual understandings. Drawing on both and integrating them are important in finding and expressing one’s personal truth, which is crucial to patient engagement and qualitative research.

As a research approach, a *Debwewin* journey is a fluid, personal, subjective methodology that uses flexible methods to complete or connect the two circles of knowledge (Gehl, 2012). Appropriate methods are needed to connect the heart and the mind. Choice of method depends on (a) gifts bestowed by the Creator, (b) context, and (c) research purpose. Gehl (2012) writes about how people can “complete, connect, and express their *Debwewin*” in diverse ways, including through artistic processes (p. 61). In our work, two research team members were trained in digital storytelling and our context and purpose fit well with digital storytelling. We believe digital storytelling was a meaningful method for facilitating a *Debwewin* journey because it attended to all four aspects of life (including emotions/heart and the intellect/mind) and integrated them in the arts-based workshop processes (Gehl, 2012). The arts are well known for providing an emotional language, and making connections between the cognitive and emotions are at the core of creative processes (Rieger et al., 2019). Gehl (2012) also writes that spaces for introspection are key for completing the circle of knowledge. The digital storytelling workshop processes provided a space for deep introspection, personal interpretation, and group sharing and bearing witness, which resulted in deep insights about patients’ experiences. We believe that the expressive arts process in the digital storytelling workshop provided a potent space and method for connecting the circle of the heart and the circle of the mind for First Nations women with experiences of cancer and fostered deep introspection and personal interpretation.

This journey can start in either circle or happen simultaneously (Gehl, 2012). Based on these participants’ stories, it appears that, with cancer experiences, people may begin the journey with their mind knowledge and, through the digital storytelling workshop processes, integrate their complex feelings to move toward a more holistic understanding rooted in the connection between the heart and mind. This need for balance of emotions and the intellect for holistic knowing is congruent with the tenets of the Medicine Wheel, which assert that balance is needed for well-being. This integration of emotions is critical in cancer care, a clinical area in which patients report an intense focus on their physical well-being at the expense of their psychosocial well-being, leaving many people with unresolved emotions and trauma from their cancer experiences (Bultz & Carlson, 2005). When this suppression of emotions is layered with other intersecting factors, such as racism and sexism, the experience of cancer can be emotionally traumatic for First Nations women. Thus, attention to safe and appropriate engagement methods becomes even more important.

The *Debwewin* journey results in a personal and holistic understanding of phenomenon. This subjective knowledge about one’s own experience is what is needed in patient engagement. As opposed to observing the one true external reality, in patient engagement we seek to understand our patients’ personal experiences of health and illness and how multiple factors intersect to profoundly, and often traumatically, shape their experiences (Gehl, 2012). The digital storytelling workshop allowed all of us to come to a holistic understanding that integrated both thoughts and emotions about First Nations women’s experiences with breast cancer and of the actual workshop, and provided insight into the intersecting factors influencing their experiences. Thus, the digital storytelling workshop facilitated a *Debwewin* journey for all involved as we came to understand patients’ experiences in a way that connected the heart and the mind (Gehl, 2012). We believe this holistic understanding is key to meaningful patient engagement and to patients’ personal truth being authentically expressed and heard in research processes.

## Further Reflections and Future Work

We found that a digital storytelling workshop can be a decolonizing method to engage *with* and learn from patients who have often been silenced in the past (Liebenberg et al., 2017). Hammond et al. (2018) asserts that an Indigenous research agenda should aim to “decolonize, transform, mobilize, and heal” Indigenous communities (p. 1). The digital storytelling workshop aligned with these goals and empowered Indigenous patients to speak about what and how new knowledge should be developed. Postcolonial feminist scholars (Anderson et al., 2009; Reimer-Kirkham & Anderson, 2002, 2010) write about the need for alternative epistemologies and methods, which can gather contextualized knowledge from those often silenced, to learn about their “material existence, rather than essentialized knowledge about people’s cultures” (Anderson et al., 2009, p. 285). Arts-based approaches, such as digital storytelling, can help us to hear the voices of marginalized people and develop this contextual, nuanced knowledge. Through understanding people’s particular stories, we can move toward more general knowledge by linking their insightful narratives to multilevel factors influencing health care experiences and inequities (Anderson et al., 2009; Reimer-Kirkham & Anderson, 2010).

The digital storytelling workshop created an ethical and relational space for patient engagement. Greenwood et al. (2017) write about the importance of creating ethical spaces to actualize cultural safety and how Two-Eyed Seeing (Martin, 2012) guides us to honor different knowledges, whereas an ethical space provides a respectful platform for this type of dialogue (Greenwood et al., 2017). An ethical space is defined as an active, energetic, process-oriented, safe space “of innovation and creativity in which Indigenous and non-Indigenous partners can come together to vision a better life now for future generations” (p. 187). The processes of the digital storytelling workshop and resulting transformation of the traditional researcher–participant relationship provided a platform in which people could enter into an authentic, holistic, and respectful dialogue about breast cancer experiences and future research (Greenwood et al., 2017). Respectful relationships are at the heart of a digital storytelling workshop and provide a vehicle for what Bird-Naytowhow et al. (2017) describe as a “ceremony of relationship.” In a scoping review of arts-based research (*N* = 36) with Indigenous peoples (Hammond et al., 2018), researchers found that artistic approaches supported respectful relationship building with participants. We believe this project demonstrates the promise of arts-based tools for providing culturally safe patient engagement and research approaches to amplify Indigenous voices (Greenwood et al., 2017)

Digital storytelling provided an engaging and empowering artistic medium for this workshop; however, the impact of using a digital medium on the ancient traditional practices of oral storytelling need to be considered. Palacios (2012) argues that the two have similar purposes: “like its mother, traditional oral storytelling, digital storytelling can foster liberation from the dominant socio-cultural world that continues to marginalize the marginalized. By creating the digital story, the storyteller has control over what is important to tell” (p. 47). Furthermore, Morgan and colleagues (2014) propose that this infusion of new mediums with ancient traditional cultural activities allows for a type of artistic resistance in which First Nations peoples can uphold their culture while engaging with Western artistic mediums. Thus, digital storytelling becomes a site of resistance and results in a shifting of the imbalance of power “so that Western culture is the one being adapted and consumed” (Morgan et al., 2014, p. 571). This shift in power also enables First Nations people to use Western technology in a way that benefits their community instead of bringing it harm. Like all mediums, it changes the expression and communication of the story, perhaps to be less animated and interactive due to the stagnate nature of video. However, at the same time, it expands the reach of the individual’s story and can thus amplify the storyteller’s voice in new ways (Morgan et al., 2014).

Digital storytelling also holds potential as a method for research with other cultural minorities and, more broadly, for exploring people’s lived experiences of illness. In our ongoing funded review of digital storytelling as a method in health research (Rieger et al., 2018), several included studies were conducted with non-Indigenous participants with diverse cultural backgrounds, including African, Caribbean, and Hispanic/Latino population groups and the included studies explored various experiences of health and illness (e.g., mental health, men’s health, and women’s health). One reason digital storytelling holds potential for illuminating diverse participants’ experiences is that it is multimodal in nature. Participants express themselves through creative writing, photos, music, and other multimedia material. Each medium resonates in a unique way with the physical, emotional, and mental aspects of a person, and this switching between mediums can heighten self-awareness and elicit rich reflections of illness experiences (Malchiodi, 2018; West et al., 2020). Malchiodi (2018) writes of how the expressive arts offer three distinct ways of knowing, which uniquely support deep processing and expressing of life’s experiences: implicit knowing (revealing hidden, sensory memories/thoughts), embodied knowing (connecting to the senses/physical body enables one to know one’s inner world), and relational knowing (fostering trust with others through the affective language of the arts). In knowledge translation, the power of digital storytelling lies in its ability to capture participants’ lived experiences in an evocative, visceral, and affective manner (Rieger & Schultz, 2014). Digital stories can be used as a narrative intervention with structurally disadvantaged populations and disrupt dominant narratives to promote culturally safe care and inform policy to address health disparities (Krause & Gubrium, 2019).

There were several notable challenges in our digital storytelling workshop and identifying these has been useful for refining this approach for future research. The 2-day workshop format felt rushed on the second day, especially given our rich conversations that needed time and space. In future studies, we would plan at least 3 days for a workshop to support relationship building and Indigenous ceremony, including land-based activities, and still have adequate time for individual story creation. There were technological challenges that we will address in future work and this discovery of technological issues demonstrates the importance of pilot work within a specific context to identify challenges. There were also many times in our workshop when having knowledge of local First Nations culture was critical to navigating relationships, which reinforced the importance of having Indigenous scholars and community members as team members.

Digital storytelling presents unique ethical challenges that were described by Gubrium and colleagues (2014) and echoed in our work, for example, fuzzy boundaries between digital storytelling as a psychosocial intervention and a research method in cancer care, recruitment and consent to participate with individuals who have experienced trauma, the level of involvement of the facilitator in shaping participant’s narratives when editing stories, possible misrepresentation of Indigenous communities or reinforcement of stereotypes, the challenge of confidentiality with very distinct stories and when sharing publicly, and ownership and control of Indigenous stories, especially given the long history of colonization (Gubrium et al., 2014). Being aware of these ethical considerations will allow us to thoughtfully plan our next research project.

Although the digital storytelling workshop seemed to facilitate authentic engagement, there are some limitations with our work. We did not formally and confidentially evaluate the workshop from the participants’ perspectives, and this article is a methodological discussion of the researchers’ reflections surrounding the workshop, as opposed to a rigorous research report. In addition, we did not have ethical permission to share what we learned about First Nations women’s experiences with breast cancer, which limits the ability of the reader to evaluate the potential and value of digital storytelling methods. Finally, when considering this approach for different contexts, one must remember that our participants were from distinct First Nations groups and that our observations should not be applied, without consultation and thoughtfulness, to other First Nations groups on Turtle Island.

Based on the feedback that we received during our digital storytelling workshop, and the findings of a systematic review on digital storytelling (Rieger et al., 2018) as a method in health care research, we are embarking on a new research project together. The early collaboration during our digital storytelling workshop has helped us to develop relevant, acceptable, and feasible research plans guided by direct input from our patient partners, and to move forward together with a Two-eyed Seeing approach. The purpose of this study is to identify and examine how storytelling has been used as a method within Indigenous health research (Rieger et al., 2020). This second study is the next step in our work together and will lead to future studies in which we employ digital storytelling as a qualitative research method to explore First Nations peoples’ experiences of cancer and other health and illness experiences.

## Conclusion

A digital storytelling workshop proved to be an innovative and meaningful approach to patient engagement in Indigenous health research and holds much potential as a decolonizing research method. Through the arts-based workshop processes, we attended to the mental, emotional, spiritual, and physical aspects of life. This holistic approach facilitated a *Debwewin* journey, and was guided by the Medicine Wheel framework by which participants were able to authentically share their complex breast cancer experiences and thoughts and feelings regarding future qualitative research. As a team, we combined our Indigenous and Settler perspectives, resulting in the strengthening of relationships and meaningful connections with the women beyond the workshop. Taking a Two-Eyed Seeing approach supported cultural safety for Indigenous cancer survivors. Through the exercise of cultural humility, we collectively learned from the participants how to meaningfully engage with them through digital storytelling methods. Continued efforts are needed to develop participatory, arts-based approaches, which can facilitate more respectful and reciprocal research practices in health care with Indigenous peoples and other cultural minorities.

## Supplemental Material

sj-pdf-1-qhr-10.1177_10497323211027529 – Supplemental material for Digital Storytelling as a Patient Engagement and Research Approach With First Nations Women: How the Medicine Wheel Guided Our Debwewin* Journey Click here for additional data file. Supplemental material, sj-pdf-1-qhr-10.1177_10497323211027529 for Digital Storytelling as a Patient Engagement and Research Approach With First Nations Women: How the Medicine Wheel Guided Our Debwewin* Journey by Kendra L. Rieger, Marlyn Bennett, Donna Martin, Thomas F. Hack, Lillian Cook and Bobbie Hornan in Qualitative Health Research
