# Supplementary material for: Digital Storytelling as a Patient Engagement and Research Approach With First Nations Women: How the Medicine Wheel Guided Our Debwewin Journey
Source: Qual Health Res. 2021 Jul 8;31(12):2163–75. doi: 10.1177/10497323211027529 (PMC8564217; doi:10.1177/10497323211027529)

Engaging Women with Experiences of Breast Cancer Using Digital Storytelling

June 18-19, 2019

Day 1
09:00 - 17:00

09:00 - 09:30	Welcome and introductions
09:30 - 10:00	Opening prayer and overview of workshop
10:00 - 10:30	Introduction to digital storytelling
10:30 - 10:45	Break
10:45 - 12:00	First talking circle
12:00 - 13:15	Break, lunch provided
13:15 - 15:00	Participants work on their story
15:00 - 15:15	Break
15:15 - 16:00	Participants record stories, and identify pictures and music
16:00 - 17:00	Introduction to the iPad app and participants work on videos

Day 2
09:00 - 17:00

09:00 - 10:30	Second talking circle
10:30 - 10:45	Break
10:45 - 12:00	Participants explore the app and continue working on videos
12:00 - 13:00	Break, lunch provided
13:15 - 14:45	Participants work on finalizing their videos
15:00 - 15:15	Feast, showcase and viewing of videos
16:00 - 17:00	Final discussion and feedback to inform future study
17:00	Distribution of certificates, honorarium, and closing prayer

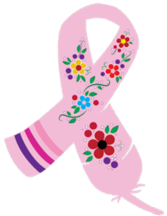

Supplement: sj-pdf-1-qhr-10.1177_10497323211027529 – Supplemental material for Digital Storytelling as a Patient Engagement and Research Approach With First Nations Women: How the Medicine Wheel Guided Our Debwewin* Journey [file sj-pdf-1-qhr-10.1177_10497323211027529.pdf]
